# Ku86 exists as both a full-length and a protease-sensitive natural variant in multiple myeloma cells

**DOI:** 10.1186/1475-2867-8-4

**Published:** 2008-04-29

**Authors:** Charles A Gullo, Feng Ge, Geraline Cow, Gerrard Teoh

**Affiliations:** 1 Department of Clinical Research (DCR), Cancer Immunology Laboratory, Singapore General Hospital (SGH), Outram Road, Singapore 169608, Singapore; 2 Multiple Myeloma Research Laboratory (MMRL), Singapore Health Services Pte Ltd (SingHealth), 7 Hospital Drive, Block A #02-05, Singapore 169611, Singapore; 3 Department of Hematology, SGH, Outram Road, Singapore 169608, Singapore

## Abstract

**Background:**

Truncated variants of Ku86 protein have previously been detected in 86% to 100% of freshly isolated patient multiple myeloma (MM) cells. Since, the Ku70/Ku86 heterodimer functions as the regulatory subunit of the DNA repair enzyme, DNA-dependent protein kinase, we have been interested in the altered expression and function of Ku86 variant (Ku86v) proteins in genome maintenance of MM.

**Results:**

Although, a number of studies have suggested that truncated forms of Ku proteins could be artificially generated by proteolytic degradation *in vitro *in human lymphocytes, we now show using whole cell immunoblotting that the RPMI-8226 and SGH-MM5 human MM cell lines consistently express full-length Ku86 as well as a 69-kDa Ku86v; a C-terminus truncated 69-kDa variant Ku86 protein. In contrast, Ku86v proteins were not detected in the freshly isolated lymphocytes as was previously reported. Data also indicates that the Ku86v was not generated as a result of carbohydrate modification but that serine proteases may act on the full-length form of the protein.

**Conclusion:**

These data confirm that MM cells contain *bona fide *Ku86v proteins that were generated intracellularly by a post-transcriptional mechanism, which required proteolytic processing.

## Introduction

Ku80 and Ku70 are two important related family members involved in the facilitation of DNA double strand break repair (DSBR) in association with the DNA repair enzyme, the catalytic subunit of DNA-dependent protein kinase catalytic (DNA-PKcs), XRCC4, DNA ligase IV and a host of other enzymes. Although present in most cells, Ku86 has been extensively studied in B and T cells due to its proposed role in the CD40-induced immunoglobulin (Ig) class switch recombination (CSR) and V(D)J lymphocyte antigen recognition/recombination events, both of which generate transient DNA double-strand breaks. The importance of this protein in lymphocyte development was most notable in Ku86 knockout mice, which failed to develop mature lymphocytes [1]. There has also been a strong association with Ku-dependent DNA DSBR and/or protection from ionizing irradiation-induced DNA damage [2-6]. Besides its well known role in DNA repair, numerous reports have implicated Ku proteins in numerous other cellular processes, including the maintenance of telomere length, regulation of G2 and M phases of the cell cycle, regulation of apoptosis and specific gene transcription, and regulation of heat shock-induced responses [7,8].

Although Ku86 and Ku70 are predominantly localized to the nucleus and the nuclear matrix, it has been found in other subcellular compartments including the cytoplasm [9] and cell membranes of numerous cell types [10,11]. The identification of Ku proteins in various compartments of the cell has led to the identification of putative novel functions of both Ku86 and Ku70. For example, in multiple myeloma (MM) cells, CD40-induced Ku86 surface expression resulted in increased cellular adhesion to fibronectin and bone marrow stromal cells [12] Other reports indicate that Ku70/Ku86 is found in the cytoplasm during mitosis and it returns to the nucleus as the cell enters the G1 phase of the cell cycle [13,14]. Finally, in colonic tumor cells, translocation of Ku86 from the cytoplasm to the nucleus occurs via interactions with a growth inhibitory tetradecapeptide and thus, Ku86 acts as a putative somatostatin receptor [15]. Therefore, location of Ku86 and other members of the DNA repair machinery in various compartments of mammalian cells may lead to numerous downstream functional consequences.

There have been a number of reports that indicate Ku86 exists in two forms, an 86 kDa full-length form and a C-terminal truncated variant form of approximately 69-kDa, in B cells from the peripheral blood (PB) [16], the acute promyelocytic leukemia (APL) cell line HL-60 [17], MM cells [5], as well as senescent fibroblasts [18]. It is unlikely that the variant protein is a product of alternative splicing of the Ku86 full length transcripts since shorter mRNAs have not been found by northern blot analysis [16,17]. Consistent with these findings, a 69-kDa variant of Ku86 was also found in the mitochondria of mammalian cells [19]. Furthermore, in the above reports, the variant of Ku86 was still able to bind DNA and associate with Ku70 consistent with the retention of the domains that are associated with those functions. Therefore, it is currently thought that variants of Ku86 are formed as a result of post-translational modification. It is the nature of this modification, which has resulted in some controversial issues regarding the physiological existence of this variant. Several recent studies have suggested that the Ku86 variant seen in lymphocytes may be due to cleavage by proteases induced during biochemical isolation [20-22]. Considering the increased amount of genomic instability seen in MM, the disregulated CD40/interleukin-4 (IL-4) pathway in MM cells [23], and the role of Ku in DNA DSBR and non-homologous end-joining (NHEJ), we investigated the presence of Ku86 and its variants in MM cells, and compared them to T lymphocytes and other cell lines. We found that unlike human T lymphocytes, the detection of 69-kDa Ku86 (Ku86v-N) variant is not likely due to *in vitro *generated protease cleavage. Moreover, we demonstrate that the full-length, as well as the truncated form of Ku86 are found in the nucleus, membrane and cytosolic fractions of resting and CD40-stimulated MM cells. Finally, we show that intracellular protease inhibition can prevent the appearance of the Ku86 variant and that the protease responsible is likely to be a serine protease. The implications for these findings are discussed.

## Methods

### Cell culture

RPMI 8226 MM, CESS Epstein-Barr virus (EBV)-transformed normal B cells, K562 chronic myeloid leukemia (CML), and HL-60 APL human cell lines were all purchased from the American Type Culture Collection (ATCC, Rockville, MD). The EBV-negative SGH-MM5 human MM cell line (CD10+ CD19- CD20- CD38+ CD40+ CD45+ CD56+ CD138+) was developed in our laboratory under the Singapore General Hospital (SGH) Institutional Review Board (IRB) good research practice guidelines, from a patient with MM using a modified Dexter-type long-term tissue culture system, which was previously described [23]. All cell lines were cultured in RPMI 1640 medium (Invitrogen, Gibco, Grand Island, NY) supplemented with 10% fetal calf serum (FCS) at 37°C and 5% CO_2_. Normal PB human T cells were isolated from buffy coat preparations from healthy donors (after informed consents were obtained) using Ficoll Hypaque density gradient centrifugation and CD3 positive magnetic bead immunoseparation (MACS columns, Miltenyi Biotec, GmbH, Gladbach, Germany). For CD40 stimulation conditions, MM cells were optimally stimulated with soluble CD40 ligand (sCD40L) (Peprotech Inc., Rocky Hill, NJ) for 4 hrs at 5.0 ng/mL [23].

### Cell extract preparation

#### Fresh whole cell extracts

Cells were washed in phosphate buffered saline (PBS), pelleted, resuspended in 2× sodium dodecyl-sulfate (SDS) loading buffer (120 mM Tris HCl pH 7.0, 4% SDS, 720 mM 2-mercaptoethanol (2-ME), 0.01% bromophenol blue and 20% glycerol), and directly boiled for 10 mins to inactivate proteases [22] as previously described. Whole cell extracts were then recovered by centrifugation at 12,000 *g *for 10 mins and loaded equally and immediately onto SDS polyacrylamide gel electrophoresis (PAGE) gels.

#### Conventional whole cell extracts

Cells were lysed in EBC1 lysis buffer (50 mM Tris HCl, pH 8.0, 150 mM NaCl, 0.1% NP-40, 50 mM NaF, 1 mM Na_3_VO_4_, 0.5 μg/ml phenylmethylsulphonylfluoride (PMSF), and 1 freshly added tablet of protease inhibitor mixture per 50 ml of buffer (Complete™ protease inhibitor tablets; Boehringer Mannheim, Roche Diagnostics GmbH, Mannheim, Germany). The mixture was then boiled in SDS sample buffer for 3 to 5 mins before loading onto SDS-PAGE gels.

#### Conventional cytosolic protein extracts

Cells were first washed in PBS and lysed in 10 volumes of the lysis buffer (10 mM Tris HCl pH 7.6, 1.5 mM MgCl_2_, 10 mM KCl, 0.5% NP-40, 1 mM dithiotretinol (DTT), and 1 freshly added Complete™ protease inhibitor tablet). Cytosolic protein extracts were recovered by centrifugation at 1,000 *g *for 15 mins [20].

#### Conventional nuclear protein extracts

From the above pellet, cell nuclei were next lysed in 5 volumes of a low-salt buffer (20 mM HEPES pH 7.9, 25% glycerol, 1.5 mM MgCl_2_, 10 mM KCl, 0.5 mM ethylenediaminetetraacetic acid (EDTA), and 1 freshly added Complete™ protease inhibitor tablet). Next, an equal volume of a high-salt buffer (i.e. low-salt buffer plus 0.8 M NaCl) was added, and the mixture left to stand on ice for 15 mins. [20]. Nuclear protein extracts were recovered by centrifugation at 16,000 *g *for 15 mins.

#### Membrane protein extracts

Cells were washed three times with ice-cold PBS, centrifuged at 3,000 *g *for 5 mins, and the pellet resuspended in 0.5 ml of TEM A lysis buffer (20 mM Tris HCl pH 8.0, 0.5 mM EDTA, 0.5 mM EGTA, 10 mM 2-ME, and 1 freshly added Complete™ protease inhibitor tablet), incubated on ice for 5 mins, and then sonicated. Membrane protein extracts were obtained by double sequential centrifugation of the lysis mixture, first at 1,000 *g *for 5 mins, then at 100,000 *g *for 30 mins for 250 μL of the supernatant. For all the assays above, Bradford's assay (Bio-Rad, Hercules, CA) was used to quantify protein concentrations in all samples.

### Western immunoblotting

Cell lysates (20.0 μg of protein/sample) were first resolved on a 12.5% SDS-PAGE gel, transferred onto polyvinylidene difluoride (PVDF) membranes (Millipore Corporation, Billerica, MA) and then blocked using Tris buffered saline Tween-20 (TBST) buffer containing 5% non-fat milk. Membranes were next hybridized overnight in the cold room using various murine monoclonal antibodies (mAb) – i.e. S10B1 anti-Ku86 N-terminus (amino acid (aa) residues 8–221; NeoMarkers, Fremont, CA); anti-heavy chain of the human major histocompatibility complex (MHC) mAb (clone 22.63.4, Accurate Chemical and Scientific Co., Westbuty, NY; a kind gift from P. Macary, from the National University of Singapore); and anti-actin (Santa Cruz Biotechnology, Santa Cruz, CA) mAbs; washed three times in ice cold TBS-T; and then incubated with horseradish peroxidase (hrp) conjugated anti-mouse IgG mAb (1:2,000; Santa Cruz) for 1 hr. The reaction was detected using the ChemiGlow chemiluminescence reagents (Alpha Innotech, San Leandra, CA). Image spot densitometry was performed on the Alpha Imager (Alpha Innotech).

### Electrophoretic mobility shift assay (EMSA)

Two 25-mer oligonucleotides; 5'-ACTTGATTAGTTACGTAACGTTATG-3' and 5'-CATAACGTTACGTAACTAATCAAGT-3', with or without biotin labels at the 5' ends (1^st ^Base Pte Ltd., Singapore), were first annealed together (see Pierce Technical Resource, TR0045.0, Pierce, Rockford, IL). Standard EMSA reactions (Lightshift Chemiluminescent EMSA kit, Pierce) incorporated 4.0 μg of cell extract and 20.0 fmol of biotin end-labeled DNA in a 20.0 μL volume binding reaction in the presence of 2.5% glycerol, 5 mM MgCl_2_, 50 ng/μL of poly(dI·dC), and 0.05% NP-40. Unlabeled target DNA (4.0 pmol) was added per 20.0 μL of binding reaction where indicated. Reactions were incubated at room temperature for 30 mins and terminated by adding 2.0 μL of 10× loading buffer (0.2% (w/v) bromophenol blue and 0.2% xylene cyanol containing 10% (v/v) glycerol). Assays were loaded onto native 5% polyacrylamide gels that were pre-electrophoresed for 60 mins in 0.5× Tris borate/EDTA buffer, resolved at 100 V, and transferred onto nylon membranes (Hybond™-N^+^, Amersham) in 0.5× Tris borate/EDTA buffer at 100 V for 30 mins. DNA was cross-linked (120 mJ/cm^2^) and detected using hrp-conjugated streptavidin chemiluminescence. Image spot densitometry was performed on the Alpha Imager (Alpha Innotech).

### Endoglycosidase H (EndoH) digestion

Endoglycosidase H resistance was assayed using the EndoH digestion system (New England Biolabs, Ipswich, MA) and performed according to the manufacturer's recommendations. Briefly, 30.0 μg of whole cell extracts were denatured at 100°C for 10 mins using 1× glycoprotein denaturing buffer, followed by addition of 1× G5 buffer and 1.0 μL (500 units) of EndoH. The reaction mix was incubated at 37°C; and at various time points, aliquots were removed and resolved in a 12.5% SDS-PAGE gel for carbohydrate (CHO) release.

### Intracellular inhibition of protease digestion

In order to inhibit intracellular protease activity, 5 × 10^6 ^cells/sample were treated with: Complete™ protease inhibitor tablets; either 1× (1 tablet for every 50 mL of media) or 2× (2 tablets for every 50 mL of media); or treated with antipain (2.0 μg/mL) plus leupeptin (2.0 μg/mL) (both from Sigma-Aldrich, St Louis, MO, USA) for cysteine protease inhibition; or aprotinin (2.0 μg/mL) plus PMSF (100 μg/mL) (both from Sigma-Aldrich) for serine protease inhibition; for to 24 hrs. Cell viability was assessed by standard trypan-blue exclusion assays. Image spot densitometry was performed on the Alpha Imager (Alpha Innotech).

### Cloning of Human Ku86 from RPMI cells

Full-length 2.4 kb Ku86 cDNA was obtained from total RNA of RPMI 8226 MM cell lines by RT-PCR (Qiagen Inc., Valencia CA, USA). The primers used to amplify the Ku86 message were forward primer: 5' -TGTATGGACGTGGGCTTTACCAT-3' and reverse primer: 5' -TCCACAGAGAATTAGATGATCCGCC-3'. Purified Ku86 cDNA (2.4 kb) was then cloned into Topo-vector (Invitrogen) and transformed into *E. coli*. The cloned Ku86 gene was fully sequenced to confirm the insertion of full length 2.4 kb Ku86 into the clone without any detected mutations (BigDye™ Cycle Sequencing Kit, Applied Biosystems, Foster City, CA USA). In order to subclone the construct into an mammalian expression system, two restriction enzyme sites were engineered to the ends of cloned Ku86 gene (*Hin*dIII at 5' end and *Xba*I at 3' end) by PCR of the clone using high fidelity PWO SuperYield™ DNA polymerase (Roche Diagnostics) and the following oligo's: Forward oligo: 5'-ATTAAAGCTTCCGGCAACATGGTGCGGTCGGGGAATAAGGCAGCTGTTGTGCTGTGTATGGACGTGGGC-3' and Reverse oligo: 5'ATTATCTAGACTTATCATGTCCAATA AATC-3'. The engineered Ku86 was then subcloned into the transient mammalian expression vector, pcDNA3.1/myc-His B (Invitrogen). The purified plasmid (Ku86+ pcDNA3.1/myc-His B) was then transfected into mammalian cell line (COS-7) for transient expression of Ku86 recombinant protein, using the Lipofectamine 2000™ reagent (Invitrogen). Finally, the Ku86 recombinant proteins were purified using ProBond™ Nickel-Chelating Resin column (Invitrogen) and detected using western Immunblotting with anti-myc-HRP (Invitrogen) and/or anti-Ku86 antibody (Neomarker).

### Trypsin digestion of recombinant human Ku86 (rhKu86) protein

Full-length rhKu86 was first expressed and purified from COS cells and digested (6.5 μg/sample or 13.0 μg/sample) using trypsin (0.065 μg of Trypsin Gold/reaction, Promega Corp. Madison, WI); at 100:1 protease:protein ratio, in 50 nM acetic acid, pH 8.0, and 37°C, as recommended at by the manufacturer (Promega Technical Bulletin, 309). The reaction was stopped by rapid freezing on ice, and analyzed using SDS-PAGE and western immunoblotting.

## Results and Discussion

### Ku86 truncation is not the result of in vitro generated proteolysis in MM cell lines

**Figure 1:**
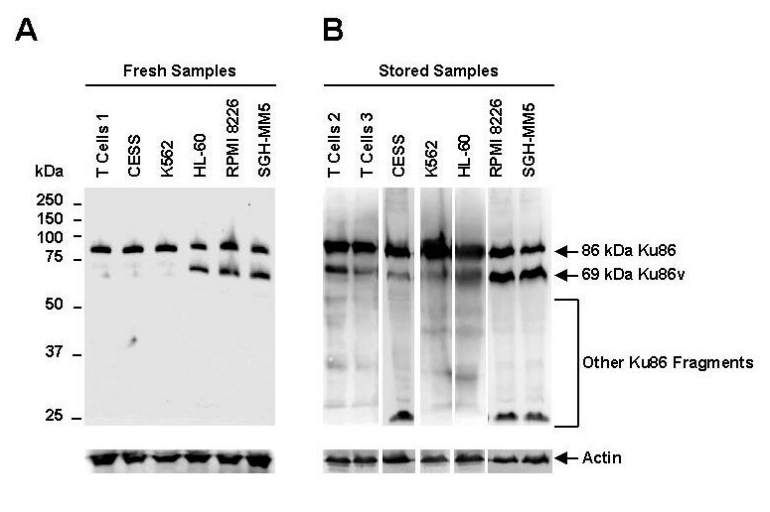
**Ku86 truncation is not the result of *in vitro *generated proteolysis in human MM cell lines**. Immediate whole cell lysis (5.0 × 10^6 ^cells/sample) was performed on freshly-obtained human PB T cells (sample from patient 1), CESS, K562, HL60, and RPMI 8226 and SGH-MM5 MM cell lines using a denaturing and reducing gel-loading buffer (95°C for 10 mins) (A). Cell extracts were also prepared using conventional methods from previously frozen and stored human PB T cells (samples from patients 2 and 3), K562 CML, and HL60, and RPMI 8226 and SGH-MM5 MM cell lines (B). Cell lysates (20.0 μg/sample) were resolved on a 12.5% SDS-PAGE gel, transferred onto PVDF membranes, and probed with S10B1 anti-Ku86 mAb, which recognizes the N-terminus of Ku86. Membranes were stripped and re-probed using anti-actin mAb (control) to confirm equal protein loading. Experiments were performed in triplicate.

Although a number of studies have characterized a 69-kDa to 70-kDa truncated variant of Ku86 *in vitro*, a few recent studies have suggested that this variant may be the result of *in vitro *induced proteolysis during storage, handling and lysis of B or T lymphocytes [21,22]. In this present study, an SDS-PAGE whole cell lysis procedure, in which all proteolytic activity is inhibited during isolation, was used to demonstrate that RPMI 8226 and SGH-MM5 MM cells (Fig. 1A, lanes 5 and 6) contain a 69-kDa N-terminus Ku86v despite the omission of the protein extraction steps and minimization of protease action. In contrast, and in agreement with prior findings, human T cells (samples from patient 1; Fig. 1A, lane 1) freshly isolated from the PB did not display altered forms of Ku86. Furthermore, CESS EBV-transformed B cell and K562 CML cell lines (Fig. 1A, lanes 2 and 3), which are known to lack the expression of the 69-kDa variant of Ku86, served as negative controls; and the HL-60 APL cell line (Fig. 1A, lane 4), which is known to contain the 69-kDa form of Ku86v, was used as a positive control for this assay. These data suggest that the 69-kDa form of Ku86v is generated *in vivo*, and is not likely to be an *in vitro *artifact, in human MM cell lines.

In order to demonstrate *in vitro *proteolysis of Ku86, we next analyzed protein extracts prepared using conventional methods from previously frozen and stored cells (Fig. 1B). Specifically, human T cells (samples from patients 2 and 3), CESS and the K562 cell lines, that lack 69-kDa Ku86v expression (Fig. 1B, lanes 1 to 4); were compared with the HL-60 cell line, which is known to express 69-kDa Ku86v, RPMI 8226, and SGH-MM5 MM cell lines (Fig. 1B, lanes 5 to 7). In this experiment, both human T cells, as well as the CESS and K562 cell lines not only contained 69-kDa Ku86v, but also various other fragments of Ku86. Moreover, even HL-60, RPMI 8226 and SGH-MM5 cell lines, that constitutively express 69-kDa Ku86v, also contained numerous Ku86 fragments. These data suggest that freezing and storage of cells leads to extensive *in vitro *proteolysis of cellular proteins. Furthermore, whole cell extracts from the RPMI 8226 cell line, which were made in a protease-free extraction buffer, i.e. either extraction buffer plus 1× Complete™ tablet (Roche Diagnostics) and 1× PMSF, or extraction buffer plus 2× Complete™ tablets and 2× PMSF, demonstrated no change in the expression of the Ku86 variant or its full-length form (data not shown). Collectively, these data confirm that although proteases released during isolation procedures can lead to extensive Ku86 degradation *in vitro*, truncated forms of Ku86 in RPMI 8226 and SGH-MM5 MM cell lines are more likely to have been generated *in vivo *and constitutively.

### The 69-kDa Ku86v in MM cell lines are present in the cytosolic, nuclear and membrane fractions and binds DNA

**Figure 2:**
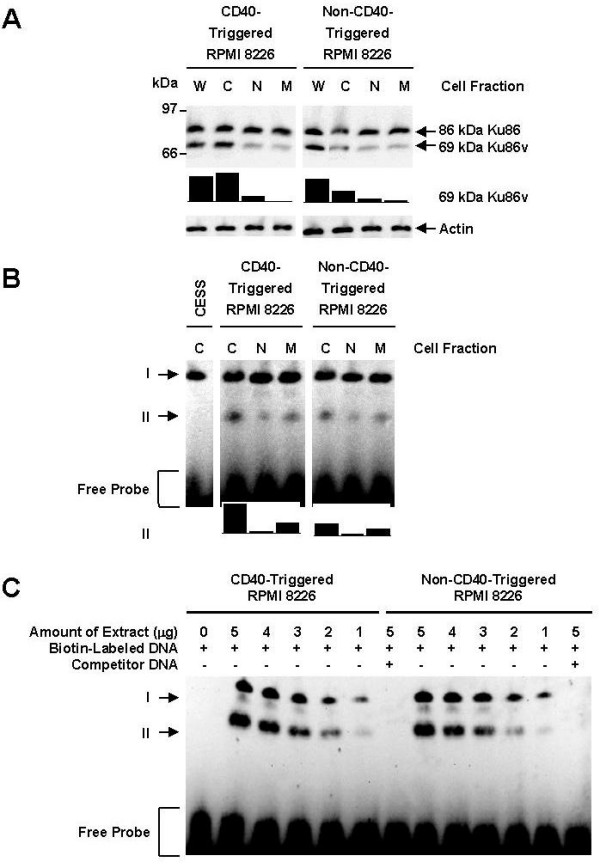
**The 69-kDa Ku86v in MM cell lines is present in the cytosolic, nuclear and membrane fractions and binds DNA**. Having determined that MM cell lines constitutively express 69-kDa Ku86v, we next studied the subcellular location of Ku86v. Moreover, since CD40 triggering induces expression of 86-kDa Ku86 on the cell membrane of MM cells, we also investigated whether CD40 triggering affected the subcellular location Ku86v. Fresh whole cell (W), cytosolic (C), nuclear (N) and membrane (M) cell lysate fractions were obtained from sCD40L-triggered (5.0 μg/mL for 4 hrs) and resting RPMI 8226 MM cells, and subjected to normal SDS-PAGE (A). Full-length and variant forms of Ku86 were detected by western immunoblotting using S10B1 anti-Ku86 mAb. Membranes were stripped and re-probed using anti-actin mAb (control) to confirm equal protein loading. Relative expression of 69-kDa Ku86v (normalized to weakest band) was determined using image densitometry and expressed in bar chart format. The presence of Ku86/Ku86v protein-DNA complexes were next detected using EMSA (B and C). Cytosolic (C), nuclear (N) or membrane (M) protein extracts (4.0 μg/sample) were first obtained from CESS (negative control), CD40-triggered (5.0 μg/mL sCD40L for 4 hrs) or non-CD40-triggered RPMI 8226 MM cell lines. Non-competitive binding of Ku86/Ku86v to DNA (B) was detected using a specific biotin end-labeled DNA probe (20.0 fmol/sample), native PAGE, and hrp-conjugated streptavidin chemiluminescence imaging. Full-length Ku86-DNA complexes are found at position I, whereas DNA complexes with the truncation variant of Ku86 are found at position II. Relative expression of 69-kDa Ku86v-DNA complexes (normalized to weakest band) was determined using image densitometry. To confirm the specificity of DNA binding, a competitive assay (C), in which a variable amount of cell lysate (up to 5.0 μg/sample) was mixed with a fixed amount (4.0 pmol/sample) of non-biotin-labeled DNA, was also performed in the same fashion. All experiments were repeated with the SGH-MM5 MM cell line (data not shown), showed similar results, and performed in triplicate.

Since CD40 activation of MM cell lines results in the membrane expression of Ku86 in MM cells [6,12,12], we also investigated the distribution of 69-kDa Ku86v in various subcellular locations, i.e. cytosol, nucleus and cell membrane, relative to CD40 triggering. As can be seen in Fig. 2A, 69-kDa Ku86v is present in all subfractions but is at higher (2.5-fold) levels in the cytosol after CD40 triggering of the RPMI 8226 MM cell line. In order to define a functional role for this observation, we analyzed the DNA-binding characteristics of Ku86 and Ku86v to a known 25-bp Ku86 binding DNA oligonucleotide using EMSAs (Figs. 2B and 2C) [16,22], in CD40-triggered RPMI 8226 MM cells in various subcellular fractions. We demonstrate binding of DNA to both 86-kDa Ku86 (band position I) as well as 69-kDa Ku86v (band position II); confirming the presence of these proteins in the RPMI 8226 MM cell line. In contrast, only full-length Ku86 was found in the negative control CESS cells. Similar results were obtained for the SGH-MM5 MM cell line (data not shown). Interestingly, no other bands are detected suggesting that if other forms of Ku86 exist they do not bind DNA. Moreover, cold competitor DNA was used to confirm specificity of the 25-bp oligonucleotide for Ku86 binding (Fig. 2C). Since the DNA binding domain of Ku86 is located in the N-terminus and is preserved in both full-length Ku86 as well as the 69-kDa Ku86v, our data not only confirms the presence of truncated Ku86v, but also suggests a functional role for Ku86v.

Surprisingly, triggering of MM cells via CD40 had no effect on the amount of 86-kDa Ku86 protein bound to DNA in all cellular subfractions (Fig. 2B, band position I) isolated from RPMI 8226 cells and well as SGH-MM5 MM cell line (data not shown). By contrast, binding of 69-kDa Ku86v to DNA was constitutively greater in the cytosol and cell membrane, but not in the nucleus (Fig. 2B, band position II); suggesting that 69-kDa Ku86v could be functionally different in different cellular compartments. Binding of DNA to 69-kDa Ku86v in both the cytosol and cell membrane is increased following CD40 triggering of RPMI 8226 MM cell lines; suggesting that CD40 triggering could induce the DNA-binding activity of Ku86v. This increased binding following CD40 triggering is not accompanied by increased amount of Ku86v expression in the nuclear fraction, as the levels are similar before and after CD40 triggering (Fig. 2A). Whereas this appears to be true for cell membrane Ku86v, expression of 69-kDa Ku86v in the cytosol is increased by approximately 2.5-fold after CD40 triggering (Fig. 2A), suggesting that increased sequestration or recruitment of 69-kDa Ku86v to the cytosol, rather than an increase in the DNA-binding activity of Ku86v, could be the more probable reason for these observations. This data is in contrast to a previously published report that shows a predominant Ku86 variant DNA-binding complex in nuclear extracts of human lymphocytes [20] and suggests that MM cells have a unique pattern of Ku86 distribution.

### 69-kDa Ku86v in MM cell lines is not the result of alternative splicing of RNA or post-translational modification by N-linked deglycosylation

**Figure 3:**
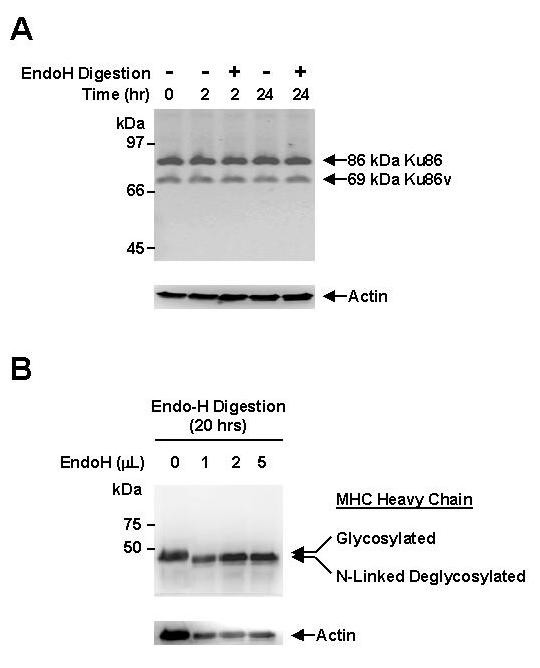
**69-kDa Ku86v in MM cell lines is not the result of post-translational modification by N-linked deglycosylation**. Freshly obtained cell lysates (30 μg/sample) from the SGH-MM5 MM cell line were incubated with or without *Endo*H (500 U/reaction) for 2 hrs or 24 hrs (A); or with varying amounts (0, 1, 2, 3, 4 or 5 μL) of *Endo*H (500 U/μL) for 20 hrs (B; positive control); and then resolved in a 12.5% SDS-PAGE gel, and immunoblotted using S10B1 anti-Ku86 (A), or anti-heavy chain of the human MHC class I protein mAbs (B). Membranes were stripped and re-probed using anti-actin mAb (control) to confirm equal protein loading. Experiments were performed in triplicate.

Since 69-kDa Ku86v in MM cell lines does not result from proteolytic cleavage during cell lysis, we asked whether the RNA transcript arose through alternative splicing. As was reported in other studies, [5,16] northern blotting demonstrated only two RNA transcripts of 3.4 kb and 2.6 kb length in RPMI 8226 and SGH-MM5 MM; K562 (negative control); and HL-60 (positive control) cell lines (data not shown). Moreover, the larger 3.4 kb RNA transcript contains both the 5' and 3' regulatory untranslated material [24,25] as well as the typical translated product. These data concur with previously published data and suggest that both RNA transcription and splicing are normal in RPMI 8226 and SGH-MM5 MM cell lines. Moreover, CD40 activation did not appear to affect RNA transcription and splicing of Ku86 (data not shown). Since post-translational modifications can also generate truncated protein variants, and numerous proteins in MM cells have been found to be heavily glycosylated [26-28], we studied the post-translational carbohydrate modifications of Ku86 and Ku86v proteins in order to determine whether the 69-kDa Ku86v is the result of post-translational degycosylation. As can be seen in Fig. 3A, neither short term (2 hrs) nor long term (24 hrs) *Endo*H treatment resulted in a decrease of 69-kDa Ku86v and concomitant increase of 86-kDa Ku86; suggesting that truncated Ku86v was not the result of N-linked deglycosylation of the 86-kDa Ku86 [29]. By contrast, *Endo*H treatment induced a faster migrating form of the 44-kDa heavy chain of the human (MHC) class I protein (Fig. 3B), which is known to be heavily glycosylated in the endoplasmic reticuculum [30,31].

### Proteolysis plays an important role in the intracellular generation of 69-kDa Ku86v in MM cell lines

**Figure 4:**
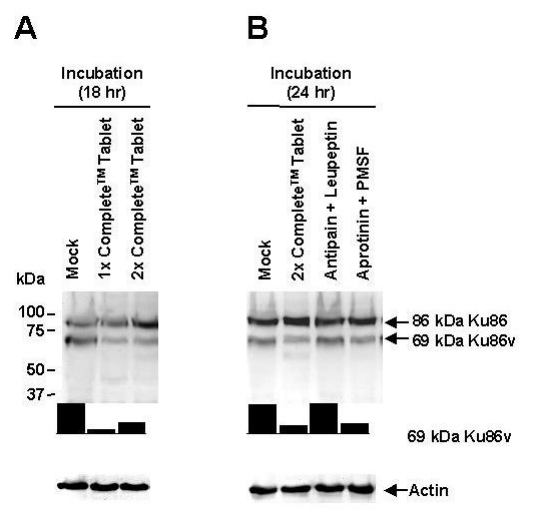
**Proteolysis plays an important role in the intracellular generation of the 69-kDa Ku86v variant protein in MM cell lines**. RPMI 8226 (A) and SGH-MM5 (data not shown) MM cell lines (5.0 × 10^6 ^cells/sample) were first incubated for 18 hrs with either 1× or 2× Complete™ protease inhibitor tablets (lanes 2 and 3); and then washed and checked for viability using trypan blue exclusion assay. Mock experiments in which cell lines were incubated for 18 hrs with media alone (lane 1) served as negative controls. Cell lysates were resolved by SDS-PAGE and immunoblotted using S10B1 anti-Ku86 mAb. The RPMI 8226 MM cell line s (B) was also incubated for 24 hrs with 2× Complete™ protease inhibitor tablets (lane 2), antipain (2.0 μg/mL final concentration) plus leupeptin (2.0 μg/mL final concentration) (lane 3), or aprotinin (2.0 μg/mL final concentration) plus PMSF (100 μg/mL final concentration) (lane 4) and analyzed in the same way as above. Mock experiments in which cell lines were incubated for 24 hrs with media alone (lane 1) again served as negative controls. Membranes were stripped and re-probed using anti-actin mAb (control) to confirm equal protein loading. Cell lysates were resolved by SDS-PAGE and immunoblotted using S10B1 anti-Ku86 mAb. Relative expression of 69-kDa Ku86v-DNA complexes (normalized to weakest band) was determined using image densitometry. All experiments were performed in triplicate.

In order to investigate other post-translational modifications that might be responsible for the generation of 69-kDa Ku86v, we looked at the intracellular proteolytic degradation of Ku86. Since our current data (Fig. 1B and 2A) have demonstrated that various *in vitro *protease inhibition strategies using fresh cell lysates did not reduce the generation of 69-kDa Ku86v, we investigated the effects of various inhibitors in whole MM cells. Within the MM cell, the ubiquitin-proteasome pathway is amongst the most active intracellular protein degradation systems. Surprisingly, incubation of whole MM cells with 2 to 24 ng/mL of bortezomib (Velcade^®^, Millennium Pharmaceuticals, MA, USA), a proteasome inhibitor, for as much as 48 hrs, had no effect on proteolytic generation of Ku86 or Ku86v (data not shown); suggesting that other proteolytic systems could be active in the MM cell. However, simultaneous inhibition of several classes of proteases; namely serine, cysteine and metallo- proteases using Complete™ tablets for 18 hrs was associated with 3- to 7-fold decreased expression of 69-kDa Ku86v (Fig. 4A lanes 2 and 3), as compared to the mock experiment (Fig. 4A lane 1). The data again suggest that 69-kDa Ku86v is generated constitutively within living MM cells. Similarly, broad-based inhibition of serine proteases via aprotinin plus PMSF (Fig. 4B lane 4), but not inhibition of cysteine proteases via antipain plus leupeptin (Fig. 4B lane 3), for 24 hrs is associated with 3- to 4-fold decreased expression of 69-kDa Ku86v; further suggesting that serine proteases (but not cysteine proteases) could be involved in generation of 69-kDa Ku86v. Importantly, protease inhibition did not affect cell viability (data not shown), or expression of 86-kDa Ku86 or actin. These data are consistent with a report that suggests that nuclear serine proteases are responsible for *in vitro *generated forms the Ku86 variant [22].

### Trypsin generates a 69-kDa Ku86 variant protein from a full-length cloned human MM Ku86 protein (rhKu86) *in vitro*

**Figure 5:**
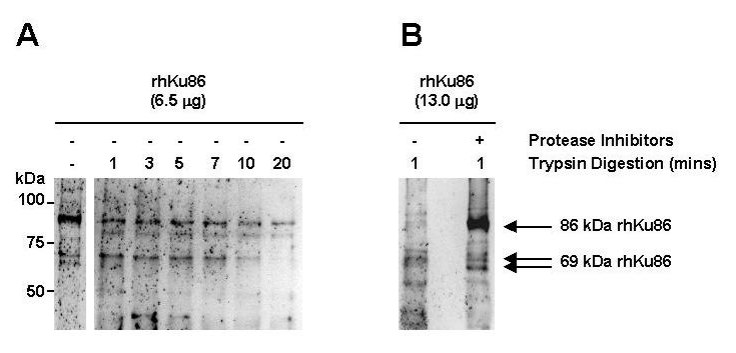
**Trypsin digestion generates a 69-kDa Ku86 protein from a full-length cloned human MM Ku86 protein (rhKu86) in vitro**. Full-length rhKu86 was first expressed and purified from COS cells. Serial digestion (A) of rhKu86 (6.5 μg/sample) by trypsin (0.065 μg of Trypsin Gold/reaction up to 20 mins) was then performed using a limited proteolysis protocol from the manufacturer. The reactions were stopped by rapid cooling on ice; and the products of trypsin digestion were resolved by SDS PAGE and immunoblotted S10B1 anti-Ku86 mAb. The effect of protease inhibitors aprotinin (2.0 μg/mL final concentration) plus PMSF (100 μg/mL final concentration) on trypsin digestion was also determined on a larger sample of rhKu86 (13.0 μg/sample). Trypsin digestion (0.065 μg of Trypsin Gold/reaction for 1 min) of rhKu86 was performed as above either without (lane 1) or with (lane 2) protease inhibitors (B).

In order to specifically test whether the 69-kDa Ku86v protein could be a result of protease digestion of full-length 86-kDa Ku86, we first cloned full-length human Ku86 from MM cell lines and produced full-length recombinant human Ku86 (rhKu86) in COS cells. During the purification of the 6-histadine-tagged rhKu86 protein from COS cells complexes of around 220-kDa were observed in the flow through and whole cell extracts, but in the eluted concentrated rhKu86 protein fraction, no higher order complexes were observed (data not shown). This suggests that most of the Ku86 protein does not associate with monkey Ku70 and is likely due to the large excess of cloned human Ku86 to that of the endogenous monkey Ku70 proteins. Thus, this synthetic protein is likely to exist as homodimer, lacks stabilization by its usual molecular partner, Ku70, and probably undergoes some spontaneous degradation in this expression system (Fig. 5A lane 1). As can be seen in Fig. 5A lane 2, trypsin digestion rapidly (within the first minute) reduced the amount of 86-kDa rhKu86 and increased the proportion of a 69-kDa Ku86 fragment. By the third minute (Fig. 5A lane 3) other smaller fragments of Ku86 have also appeared. Digestion continues over several mins, and by 20 mins., almost the entire original 86-kDa rhKu86 as well as the 69-kDa proteins have been digested (Fig. 5A lanes 4–7). These data suggest that trypsin-like proteases could preferentially generate a 69-kDa fragment of Ku86 protein. Accordingly, to determine if protease inhibitors could now inhibit the generation of this protein, we performed trypsin digestion of rhKu86 without (Fig. 5B lane 1) or with (Fig. 5B lane 2) protease inhibitors (aprotinin plus PMSF). We demonstrate that protease inhibition is very effective in inhibiting breakdown of 86-kDa rhKu86; but as expected, protease inhibition is not complete and some smaller fragments are formed. However, the further degradation of Ku86 is virtually inhibited. These data confirm that trypsin-like activity is likely to contribute (at least in part) to the degradation of Ku86 into a 69-kDa variant form.

## Conclusion

Karyotypic analysis of tumor cells from patients with MM, as well as MM cell lines, frequently demonstrates numerous complex chromosomal abnormalities. Moreover, new chromosomal translocations into the switch region of the immunoglobulin heavy chain (IgH) gene (chromosome 14q32) often heralds transformation to more aggressive MM [23] Since DNA DSBR is important in mediating these processes, this suggests that abnormalities in DSBR could ultimately lead to genomic instability, clonal evolution and disease progression in MM. Truncated variants of Ku86 protein (i.e. Ku86v) have previously been detected in 86% to 100% of freshly isolated patient MM cells [5]. However, since the expression of an altered form of the Ku86 protein in B cells [16] has been recently challenged [21]. as perhaps being non-physiological, we carefully investigated the presence of truncated forms of Ku86. The presence of the 69-kDa truncated form of Ku86 detected by antibodies directed against the N-terminus part of the protein was confirmed in both traditional western blots as well as direct lysate fresh whole cell western immunoblotting.

In agreement with previously published data that the C-terminus truncated Ku86 variant from MM cells binds double stranded DNA ends [5], we show DNA end-binding (DEB) activity with both the full-length and truncated variant of Ku86 in all cellular subfractions tested. This is consistent with the 69-kDa variant of Ku86 that was found in mammalian mitochondria that still associates with DNA and is found in a complex with Ku70 [19]. Since the DNA binding motifs of Ku86 are located in the N-terminus, and the functional domains are located in the C-terminus, these data support the notion that while Ku86v-C binds DNA, it is in fact incapable of regulating DNA repair. This is further supported by published data that demonstrated the heteroduplex of Ku70 and Ku86 variant bind DNA-PKcs less well than those containing full-length Ku86 [5] and are deficient in DNA-PK activity [16]. The link between the defective DNA-PK activity as a result of Ku86 variant and Ku70 heteroduplex formation and genomic instability in MM has not been proven thus far, although cells expressing Ku86 variants do display increased sensitivity to DNA damage [5,16,17]. The ability of multiple myeloma-specific Ku86 variant protein to bind Ku70 or itself and to activate DNA-PK and its overall effect on DNA repair in MM cells is the subject of ongoing work.

Since protease digestion of DNA-PK and Ku proteins are enhanced by proteasome inhibition (i.e. bortezomib treatment) in MM cell lines [32] these data taken in aggregate further suggest that proteolytic enzymes that are capable of digesting Ku proteins are constitutively activated, and possibly accumulate and/or become further activated under proteasome inhibition in MM cells. Thus, the observed trypsin-like serine protease-dependent cleavage of Ku86 during the isolation procedures [22] may in fact operate naturally in intact MM cells. These findings, as well as those that found that modification of Ku86 can occur with viral infections, phorbyl ester, and calcium phosphate treatment in CV1 cells [33], suggest that under certain conditions or in certain cells lines Ku86 truncation may occur in a non-inducible proteolytic-dependent fashion. Our data suggests that these potential proteases may be acting in the cell naturally and perhaps in a constitutive fashion in MM cells. Serine protease inhibition is much more effective than cysteine protease inhibition in reducing the appearance of the Ku86 variant in whole RPMI cells (Fig. 4B). Using rhKu86 and *in vitro *limited digestion, one likely candidate may be trypsin (Fig. 5). In fact, by looking at the sequence of Ku86 and using bioinformatics approach we were able to narrow down potential digestion of Ku86 into 20 different proteases (using the Peptide Cutter Peptide Characterization software- ExPASy, Swiss Institute for Bioinformatics). Trypsin, chymotrypsin and other serine proteases putatively cleave Ku86 in multiple locations. However, finding the exact physiologically relevant protease that generates the variant *in vivo *will require more detailed analysis. We have only partially attempted to address this by using protease inhibitor in living cells. At the doses used, antipain and leupeptin are more selective towards cysteine inhibition than towards inhibition of serine proteases and aprotinin and PMSF are powerful serine protease inhibitors. However, it is important to note that there is likely to be some cross-inhibition. Thus, other studies using various single and multiple cysteine and serine protease inhibitors as well as mettaloproteinase inhibitors such as TIMP-2, will be needed to more closely address this issue. However, this study as well as two others, [18,22] suggest that serine proteases are likely to be important for the generation of the 69-kDa form of Ku86. However, our studies as well as others investigate Ku86 and its variant in conditions that do not allow for the association with Ku70. It is well known that Ku86 is most effective in binding DNA and recruiting DNA repair enzymes when it is heterodimerized with Ku70 which occurs via the C-terminus of both proteins [34]. We are interested in determining the naturally occurring proteolytic cleavage site of Ku86 and to examine how it affects hetero-complex formation. We hypothesize that a fraction of newly synthesized Ku86 protein is immediately and constitutively cleaved by proteases into a Ku86 69-kDa form that would prevent it from tight association with Ku70.

The localization of Ku86 within different compartments within the cell is likely to be of significant importance in B cell DNA repair and perhaps in the regulation of genomic stability of MM cells. In fact, the intracellular trafficking of Ku protein to extranuclear sites has been suggested to play important role in numerous non-DNA repair Ku-associated functions [7,13] Importantly, the localization of Ku to the membranes of MM cells following sCD40L treatment leads to increased adhesion to bone marrow stromal cells and interneukin-6 (IL-6) production [12]. These and other studies prompted us to thoroughly examine the localization of Ku86 and Ku86 variant in the nucleus, cytosol and membrane of MM cells before and after CD40 stimulation. Unexpectedly, there were little differences observed in each of the cellular fractions for the full-length Ku86 protein levels in MM cells. Contrary to previously published data that shows increased Ku86 truncation variant in the nuclear fraction of human PBMC [22], we found equal levels of the Ku86v in the membrane, nuclear and cytosolic fractions in unstimulated MM cells. Yet, when the cells were stimulated with sCD40L, we did observe an increased amount of Ku86 variant in the cytosol accompanied by an increase in the DNA binding activity of Ku86v in the cytoplasm and not in the nucleus. These findings may be due to the cell type or to the fact that there appears to be little to no *in vitro *generated protease cleavage of Ku86 occurring during our biochemical preparations. However, the nature of the truncation is still unknown and unlikely due to post-transcriptional modification or carbohydrate modification. We are currently using more sensitive techniques of isolating various cellular fraction, performing 2-dimensional gel electrophoresis, and mass spectrometry, as well as Edmund degradation to try and determine the exact nature of this MM-specific Ku86 truncation variant. We are also currently performing studies to determine the biological function of the variant and how it might interact or disrupt the normal Ku86/Ku70/DNA-PKcs holoenzyme. Finally, we routinely see both the full-length and the truncation variant in all of our cell fractions and have not seen a time-dependent reduction in the amount of full-length Ku86 nor an increase in the variant Ku86 under any conditions. We therefore believe that the variant seen in MM cell lines is regulated in a distinct fashion from the Ku86 truncation variant that is seen in PBMCs.

## Abbreviations

CSR: Class switch recombination; DNA-PKcs: DNA-dependent Protein Kinase catalytic subunit; DSBR: DNA Double Stranded Break Repair; Endo H: Endonuclease H; MM: Multiple Myeloma; NHEJ: Non Homologous End Joining.

## Competing interests

The authors declare that they have no competing interests.

## Authors' contributions

FG and GC performed all of the molecular and biochemical experiments in this manuscript. CG and GT conceived of the study, and participated in its design and coordination and helped to draft the manuscript. All authors read and approved the final manuscript.

## References

[B1] Nussenzweig A, Chen C, da CS, Sanchez M, Sokol K, Nussenzweig MC, Li GC (1996). Requirement for Ku80 in growth and immunoglobulin V(D)J recombination. Nature.

[B2] Lieber MR, Grawunder U, Wu X, Yaneva M (1997). Tying loose ends: roles of Ku and DNA-dependent protein kinase in the repair of double-strand breaks. Curr Opin Genet Dev.

[B3] Muller C, Calsou P, Frit P, Cayrol C, Carter T, Salles B (1998). UV sensitivity and impaired nucleotide excision repair in DNA-dependent protein kinase mutant cells. Nucleic Acids Res.

[B4] Salles B, Calsou P, Frit P, Muller C (2006). The DNA repair complex DNA-PK, a pharmacological target in cancer chemotherapy and radiotherapy. Pathol Biol (Paris).

[B5] Tai YT, Teoh G, Lin B, Davies FE, Chauhan D, Treon SP, Raje N, Hideshima T, Shima Y, Podar K, Anderson KC (2000). Ku86 variant expression and function in multiple myeloma cells is associated with increased sensitivity to DNA damage. J Immunol.

[B6] Tai YT, Podar K, Kraeft SK, Wang F, Young G, Lin B, Gupta D, Chen LB, Anderson KC (2002). Translocation of Ku86/Ku70 to the multiple myeloma cell membrane: functional implications. Exp Hematol.

[B7] Gullo C, Au M, Feng G, Teoh G (2006). The biology of Ku and its potential oncogenic role in cancer. Biochim Biophys Acta.

[B8] Tuteja R, Tuteja N (2000). Ku autoantigen: a multifunctional DNA-binding protein. Crit Rev Biochem Mol Biol.

[B9] Bakalkin G, Yakovleva T, Hurd YL, Nussenzweig A, Li GC, Terenius L (1998). Autoantigen Ku in the brain. Developmentally regulated expression and subcellular localization. Neuroreport.

[B10] Dalziel RG, Mendelson SC, Quinn JP (1992). The nuclear autoimmune antigen Ku is also present on the cell surface. Autoimmunity.

[B11] Prabhakar BS, Allaway GP, Srinivasappa J, Notkins AL (1990). Cell surface expression of the 70-kD component of Ku, a DNA-binding nuclear autoantigen. J Clin Invest.

[B12] Teoh G, Urashima M, Greenfield EA, Nguyen KA, Lee JF, Chauhan D, Ogata A, Treon SP, Anderson KC (1998). The 86-kD subunit of Ku autoantigen mediates homotypic and heterotypic adhesion of multiple myeloma cells. J Clin Invest.

[B13] Koike M, Awaji T, Kataoka M, Tsujimoto G, Kartasova T, Koike A, Shiomi T (1999). Differential subcellular localization of DNA-dependent protein kinase components Ku and DNA-PKcs during mitosis. J Cell Sci.

[B14] Koike M, Ikuta T, Miyasaka T, Shiomi T (1999). Ku80 can translocate to the nucleus independent of the translocation of Ku70 using its own nuclear localization signal. Oncogene.

[B15] Tovari J, Szende B, Bocsi J, Falaschi A, Simoncsits A, Pongor S, Erchegyi J, Stetak A, Keri G (1998). A somatostatin analogue induces translocation of Ku 86 autoantigen from the cytosol to the nucleus in colon tumour cells. Cell Signal.

[B16] Muller C, Dusseau C, Calsou P, Salles B (1998). Human normal peripheral blood B-lymphocytes are deficient in DNA-dependent protein kinase activity due to the expression of a variant form of the Ku86 protein. Oncogene.

[B17] Han Z, Johnston C, Reeves WH, Carter T, Wyche JH, Hendrickson EA (1996). Characterization of a Ku86 variant protein that results in altered DNA binding and diminished DNA-dependent protein kinase activity. J Biol Chem.

[B18] Jeng YW, Chao HC, Chiu CF, Chou WG (1999). Senescent human fibroblasts have elevated Ku86 proteolytic cleavage activity. Mutat Res.

[B19] Coffey G, Campbell C (2000). An alternate form of Ku80 is required for DNA end-binding activity in mammalian mitochondria. Nucleic Acids Res.

[B20] Lanuszewska J, Widlak P (2004). The truncation of Ku86 in human lymphocytes. Cancer Lett.

[B21] Sallmyr A, Henriksson G, Fukushima S, Bredberg A (2001). Ku protein in human T and B lymphocytes: full length functional form and signs of degradation. Biochim Biophys Acta.

[B22] Sallmyr A, Du L, Bredberg A (2002). An inducible Ku86-degrading serine protease in human cells. Biochim Biophys Acta.

[B23] Hwang WY, Gullo CA, Shen J, Poh CK, Tham SC, Cow G, Au M, Chan EW, Teoh G (2006). Decoupling of normal CD40/interleukin-4 immunoglobulin heavy chain switch signal leads to genomic instability in SGH-MM5 and RPMI 8226 multiple myeloma cell lines. Leukemia.

[B24] Cai QQ, Plet A, Imbert J, Lafage-Pochitaloff M, Cerdan C, Blanchard JM (1994). Chromosomal location and expression of the genes coding for Ku p70 and p80 in human cell lines and normal tissues. Cytogenet Cell Genet.

[B25] Muller C, Salles B (1997). Regulation of DNA-dependent protein kinase activity in leukemic cells. Oncogene.

[B26] Belessi C, Stamatopolous K, Kosmas C (2002). Glycosylation of V region genes in follicular lymphoma as a result of the somatic hypermutation mechanism. Blood.

[B27] Farooq M, Takahashi N, Arrol H, Drayson M, Jefferis R (1997). Glycosylation of polyclonal and paraprotein IgG in multiple myeloma. Glycoconj J.

[B28] Nakamura F, Kaimori M, Takaya H, Fujita K, Suzuki N, Sakurabayashi I, Yoshioka N (1996). [Markedly elevated serum fructosamine in a non-diabetic patient with IgA-kappa type multiple myeloma]. Rinsho Byori.

[B29] Gentzsch M, Cui L, Mengos A, Chang XB, Chen JH, Riordan JR (2003). The PDZ-binding chloride channel ClC-3B localizes to the Golgi and associates with cystic fibrosis transmembrane conductance regulator-interacting PDZ proteins. J Biol Chem.

[B30] Parham P (1996). Functions for MHC class I carbohydrates inside and outside the cell. Trends Biochem Sci.

[B31] van Leeuwen JE, Kearse KP (1996). Deglucosylation of N-linked glycans is an important step in the dissociation of calreticulin-class I-TAP complexes. Proc Natl Acad Sci U S A.

[B32] Mitsiades N, Mitsiades CS, Poulaki V, Chauhan D, Fanourakis G, Gu X, Bailey C, Joseph M, Libermann TA, Treon SP, Munshi NC, Richardson PG, Hideshima T, Anderson KC (2002). Molecular sequelae of proteasome inhibition in human multiple myeloma cells. Proc Natl Acad Sci U S A.

[B33] Quinn JP, Simpson J, Farina AR (1992). The Ku complex is modulated in response to viral infection and other cellular changes. Biochim Biophys Acta.

[B34] Wu X, Lieber MR (1996). Protein-protein and protein-DNA interaction regions within the DNA end-binding protein Ku70-Ku86. Mol Cell Biol.

